# Air Pollution as a Cause of Obesity: Micro-Level Evidence from Chinese Cities

**DOI:** 10.3390/ijerph16214296

**Published:** 2019-11-05

**Authors:** Zhiming Yang, Qianhao Song, Jing Li, Yunquan Zhang

**Affiliations:** 1 Donlinks School of Economics and Management, University of Science and Technology Beijing, Beijing 100083, China; zhiming0419@126.com (Z.Y.);; 2 Center for Central China Economic and Social Development Research, Nanchang University, Nanchang 330031, China; 3 School of Economics and Management, Nanchang University, Nanchang 330031, China; 4 Department of Epidemiology and Biostatistics, School of Public Health, Medical College, Wuhan University of Science and Technology, Wuhan 430065, China; 5 Hubei Province Key Laboratory of Occupational Hazard Identification and Control, Wuhan University of Science and Technology, Wuhan 430065, China

**Keywords:** obesity, air pollution, Chinese cities

## Abstract

Chinese air pollution is obviously increasing, and the government makes efforts to strengthen air pollution treatment. Although adverse health effects gradually emerge, research determining individual vulnerability is limited. This study estimated the relationship between air pollution and obesity. Individual information of 13,414 respondents from 125 cities is used in the analysis. This study employs ordinary least squares (OLS) and multinomial logit model (m-logit) to estimate the impact of air pollution on obesity. We choose different air pollution and Body Mass Index (BMI) indicators for estimation. Empirical results show Air Quality Index (AQI) is significantly positively associated with the BMI score. As AQI adds one unit, the BMI score increases 0.031 (SE = 0.002; *p* < 0.001). The influence coefficients of particle size smaller than 2.5 μm (PM_2.5_), particle size smaller than 10 μm (PM_10_), carbon monoxide (CO), nitrogen dioxide (NO_2_), ozone (O_3_), and sulfur dioxide (SO_2_) to the BMI score are 0.034 (SE = 0.002; *p* < 0.001), 0.023 (SE = 0.001; *p* < 0.001), 0.52 (SE = 0.095; *p* < 0.001), 0.045 (SE = 0.004; *p* < 0.001), 0.021 (SE = 0.002; *p* < 0.001), 0.008 (SE = 0.003; *p* = 0.015), respectively. Generally, air pollution has an adverse effect on body weight. CO is the most influential pollutant, and female, middle-aged, and low-education populations are more severely affected. The results confirm that the adverse health effects of air pollution should be considered when making the air pollution policies. Findings also provide justification for health interventions, especially for people with obesity.

## 1. Introduction

China has achieved remarkable economic development over the past 30 years; however, an unintended consequence of this growth is its negative influence on the natural environment. For example, regional pollution incidents in China have increased, which not only influence human well-being but also threaten residents’ health. Many studies have explained the impact of air pollution on human health and the incidence of diseases such as respiratory and cardiovascular diseases [1,2,3]. Several studies focused on various pollutants in the air and found that these pollutants could negatively affect multiple systems and organs in the human body at a specific concentration, thereby having a significant impact on population mortality and susceptibility to other diseases [4,5,6]. Specifically, most recent literature paid attention to the adverse effects of air pollution on cancer and chronic diseases [7,8,9,10,11,12]. For example, Jaganathan et al. (2019) [8] reviewed the relationship between long-term exposure to fine particulate matter and cardio-metabolic diseases in developing countries; Filippini et al. (2019) [10] analyzed the association between outdoor air pollution and childhood leukemia based on meta-analysis; and Gaio et al. (2019) [11] focused on air pollution and lipid profile. A large amount of evidence depicted the adverse effects of air pollution on human health problems.

Obesity is defined by the World Health Organization (WHO) as a disease characterized by the excessive accumulation of body fat [13]. In 2019, the WHO listed air pollution and obesity among the top ten threats to human health [14]. Obesity is considered as a public health problem, leading to serious social, psychological, and physical problems [13]. Existing literature has demonstrated that air pollution is one of the major factors influencing obesity in developed countries [15,16]. Therefore, it is of considerable significance to assess the impact of air pollution on obesity. However, to the best of our knowledge, few studies focus on the relationship between air pollution and obesity in developing countries, particularly in China.

Air pollution could affect obesity from several aspects. On the one hand, air pollution could increase the risk of a number of diseases. For example, Xu et al. (2010) [17], Toledo-Corral et al. (2018) [18], and An et al. (2018) [16] found that air pollution leads to metabolic disorder, which is closely related to body weight; WHO (2018) [19] proposes that air pollution increases the possibility of cardiovascular and respiratory diseases, heart diseases, and some cancers, which, in turn, affect body weight. On the other hand, pollution could also affect people’s behavior responses that influence body weight. For example, people choose to stay indoors in days with heavier pollution and reduce time for physical activities [20,21,22,23], which, in turn, reduce calories expended and cause obesity.

Most literature on the relationship between air pollution and obesity is concentrated in developed countries [15,16]. In developing countries, especially China, there are growing concerns regarding children’s exposure to pollution and increased obesity rates. Epidemiologic evidence suggests that air pollution is a risk factor in childhood obesity [16,24,25,26,27,28,29]. Childhood obesity has emerged as a major public health problem in the United States and elsewhere [30]. Children are a special group considered to be more susceptible to epidemics and air pollution. Unlike childhood obesity, we know little about the effects of obesity from air pollution on the Chinese adult population.

The above studies provide a basis for analyzing the relationship between air pollution and obesity in adults across the country. At present, few studies specifically investigate whether air pollution leads to obesity in China. Given this lack of research, the primary aim of the study is to obtain micro-level evidence from Chinese cities to research and assess the impact of air pollution on obesity in developing countries.

## 2. Methods

### 2.1. Data

**Figure 1:**
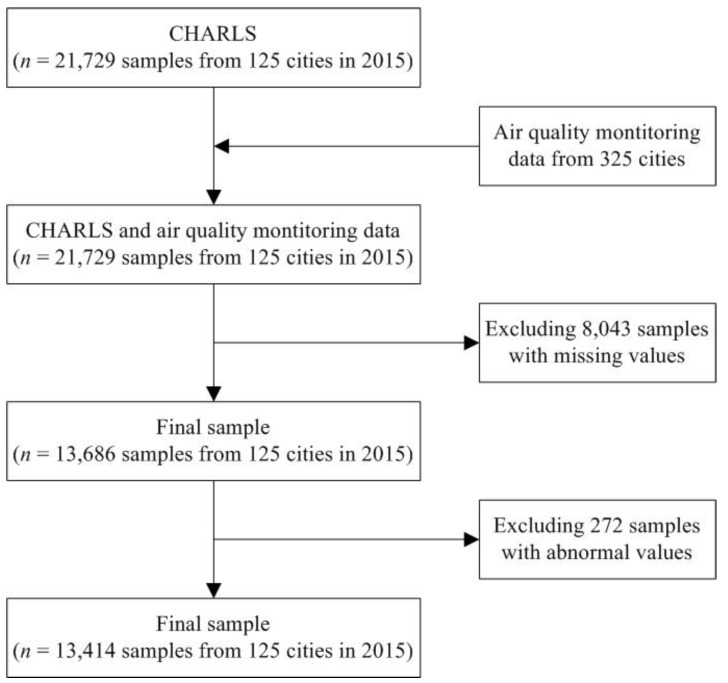
Flow chart.

**Figure 2:**
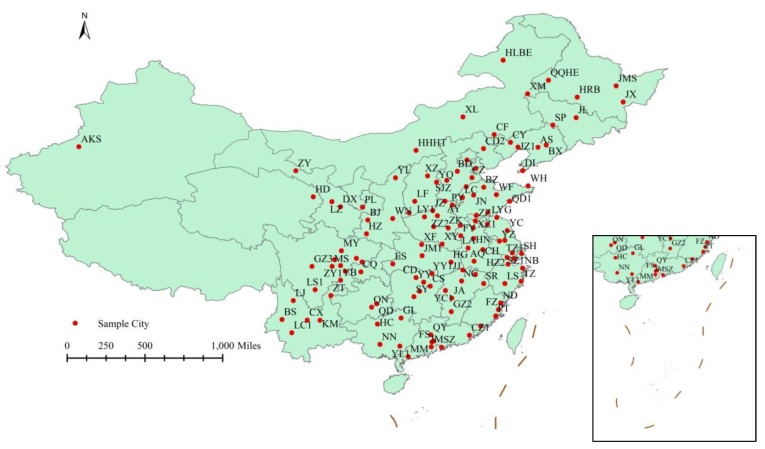
The geographical distribution of sample cities.

This study collects a dataset consisting of individual information and city-level variables to evaluate the relationship between air pollution and obesity. Individual data were derived from the China Health and Retirement Longitudinal Study (CHARLS) in 2015, which is a nationally representative survey conducted among middle-aged and elderly Chinese residents (aged 45 years and above) using face-to-face computer-assisted personal interviews. The CHARLS questionnaire included the following modules: demographics, family structure, health status and functioning capabilities, biomarkers, health care and insurance, work, retirement and pension, income and consumption, assets (individual and household), and community-level information [31,32]. The ethics committee of Peking University Health Science Center approved this study, and all participants gave written informed consent before participation [33]. Using multi-stage stratified probability-proportionate-to-size sampling, the sample in CHARLS represented approximately 12,400 households in 150 counties/districts (a total of 450 villages/resident communities). A total of 21,729 respondents participated in the interview. In this study, data from CHARLS were constructed to estimate the obesity problem and control variables. Before measurement, raw data had to be properly processed. As some respondents have missing values, 13,686 respondents in 2015 were considered. Furthermore, as the measurement error is most likely to affect the extreme value, to ensure the stability of the data and improve the accuracy and standardization of the analysis, the samples with height and weight at 1% before and after (about 272 respondents) are excluded. In the end, 13,414 respondents in 2015 are considered. Figure 1 presents a flow chart of the study process. Meanwhile, the hourly pollutant levels from 125 cities were evaluated using data from the national urban air quality monitoring network. Figure 2 presents the 125 cities in China.

### 2.2. Variables

#### 2.2.1. Body Mass Index (BMI)

Body Mass Index (BMI) is an important standard used to measure the degree of obesity and the health of the human body [13]. BMI is a relatively objective parameter to measure body mass and is calculated as weight (in kilograms) divided by the square of the height (in meters) (BMI unit: kg/m^2^). Therefore, the higher the BMI, the greater the obesity [34]. BMI as a tool for measurement is recommended by the WHO and is also widely used in epidemiological studies to assess excessive weight and obesity levels in people of different ages, sexes, and ethnicities. In addition, BMI is widely used to assess the degree of obesity and the risk of obesity-related diseases [35].

As the percentage of body fat and cardiovascular risk in Asians are usually higher for a given BMI value than in the Western population, it is recommended to establish appropriate thresholds for each country [36]. In this study, we use multiple standards, Chinese, WHO, and Asian standards, to define obesity, rather than just using one standard (see Table 1).

#### 2.2.2. Air Pollution

We obtained the hourly pollutant data from the national urban air quality monitoring network from 1 April 2015 to 31 August 2015. We primarily used the Air Quality Index (AQI) to measure air pollution. AQI is China’s new air quality evaluation standard [37] and monitors the levels of six pollutants: particle size smaller than 2.5 μm (PM_2.5_), particle size smaller than 10 μm (PM_10_), carbon monoxide (CO), nitrogen dioxide (NO_2_), ozone (O_3_) and sulfur dioxide (SO_2_). The higher the indicator values, the higher the pollution levels. According to the environmental AQI technical regulations, AQI can be divided as: 0–50, 51–100, 101–150, 151–200, 201–300, and >300, corresponding to the air quality index level 1 (excellent), level 2 (good), level 3 (mild pollution), level 4 (moderate pollution), level 5 (heavy pollution), and level 6 (serious pollution). Generally, if the air quality is excellent or good, it has less impact on outdoor activities. To test the stability of the model, we also chose other air pollution indicators for robust estimation, such as PM_2.5_, PM_10_, CO, O_3_, NO_2_, and SO_2_.

### 2.3. Estimation Strategy

Descriptive analysis is first performed to describe the sample characteristics for the total sample and by BMI according to Chinese criteria. Frequencies with percentages for categorical variables (e.g., sex, marital status, insurance status, education, health behavior, categorical BMI, and AQI levels), and means with standard deviations were reported for continuous variables (e.g., age, BMI, and air pollution indicators) were presented.

We employed the ordinary least squares (OLS) method to analyze the relationship between air pollution and the degree of obesity, which can be presented as follows:

*BMI_ic_* = *a*_0_ + *Air_ic_a*_1_ + *X_ic_a*_2_ + *e_ic_* (1)

where the subscript *i* and *c* denote individual and city respectively, *BMI_ic_* is the degree of obesity of individual *i* in city *c*; *Air_ic_* denotes the air pollution of person *i* in city *c*; *X_ic_* represents individual characteristics and health behavior of person *i* in city *c*, including sex, age, education, etc.; *a*_0_, *a*_1_, *a*_2_ are the parameters to be estimated; *e_i__c_* is the idiosyncratic error term and it obeys a normal distribution.

Given the existence of the BMI standard and the categorical variables, the OLS estimation may provide inconsistent estimates. Therefore, we chose multinomial logit model (m-logit), which can be expressed as:

BMI*_ic_ = *b*_0_ + *Air_ic_b*_1_ + *X_ic_b*_2_ + *e_ic_*

BMI*_ic_ = 0, if *BMI_ic_* <18.5

= 1, if 18.5 ≤ *BMI_ic_* < 23.9 (2)

= 2, if 24 ≤ *BMI_ic_* < 27.9

= 3, if *BMI_ic_* ≥28

where *BMI_ic_* has 4 levels: Underweight, Normal, Overweight and Obese; *b*_0_, *b*_1_, *b*_2_ are the parameters to be estimated; and BMI*_ic_ is a latent variable and *BMI_ic_* is its observable variable. In addition, we chose different air pollution and BMI indicators for estimation and examined the impact of lagging air pollution. A stratified analysis approach was used to discuss the role of sex, age, and education in air pollution and obesity. Dependent variable is BMI score, and we used OLS estimation, with robust standard errors. Dependent variable is BMI according to Chinese criteria, and we used m-logit estimation. Stata 14 was applied to statistical analysis.

## 3. Results

**Figure 3:**
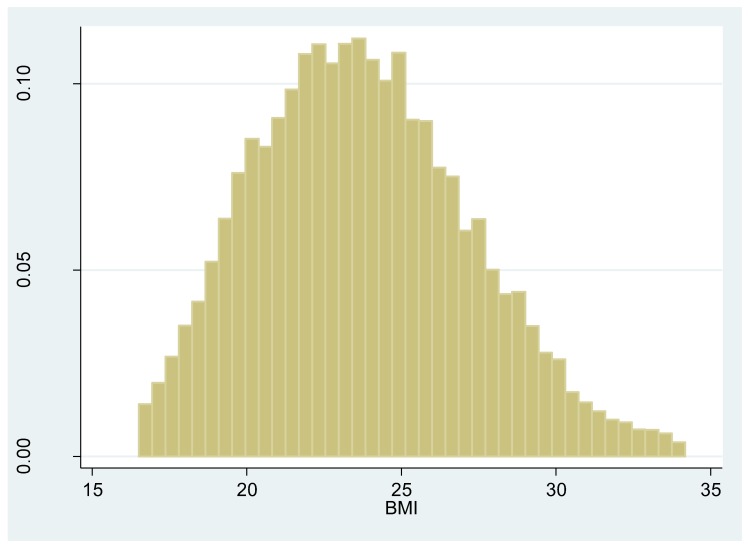
Histogram of the Body Mass Index (BMI) score.

Respondents’ characteristics and average AQI levels across BMI levels are shown in Table 2. The total study population consisted of 13,414 respondents with a mean age of 61 years; 46.7% were male, 86.64% were married, and 8.12% were uninsured. A minority, 25.70% of participants had no formal education, 19.69% did not finish primary school and home school, 22.68% elementary school, and 31.93% had middle school education and above. A total of 30.77% respondents smoked, and 26.09% reported that they drank alcohol frequently. The average BMI was 23.77 and 12.02% were obese (Meanwhile, Figure 3 depicts the histogram of the BMI score). The average monthly AQI was 64.39 and a minority, 20.8% of samples had low AQI categorical levels (<49.9); the vast majority (76.9%) of samples had moderate categorical AQI levels (50–99.9), 2.3% of samples have a higher AQI categorical levels (>100). The average continuous AQI was 67.1 when we used a one-month lag, 74.04 when we used a two-month lag, and 79.72 when we used a three-month lag.

AQI on BMI is shown in Table 3. In Table 3, the second and third columns represent the results from the OLS regression, which are the results of Equation (1). Columns 4 to 9 denote the results of m-logit, which are the results of Equation (2). Generally, Coefficients and robust standard errors are reported for the OLS regression model, while the adjusted odds ratio and 95% confidence interval values are used for the m-logit. The coefficient of AQI on BMI is 0.031 (SE = 0.002; *p* < 0.001), which is significantly positive, indicating a positive association between air pollution and obesity. This result is consistent with those of previous Chinese studies and results from the USA. As AQI added one unit, the BMI score increased by 0.031 (SE = 0.002; *p* < 0.001). Specifically, the coefficient for obese participants is higher than those who were underweight. The effects of AQI on “Normal”, “Overweight”, and “Obese” levels based on Chinese criteria were 1 percent (95% CI: 1.005, 1.015), 2.4 percent (95% CI: 1.019, 1.029) and 3.2 percent (95% CI: 1.027, 1.038), respectively.

The interpretation of other control variables is also necessary. Taking the OLS estimation results as an example, the coefficient of males on the BMI score was −0.229 (SE = 0.078; *p* = 0.003), meaning that if the respondent was a male, the BMI score was 0.229 lower than that of a female. The coefficient of age was −0.052 (SE = 0.003; *p* < 0.001), which has a significantly negative effect on BMI. With an increase in age, the BMI score gradually decreased. As the level of education increased, BMI increased. Compared with “No formal education”, respondents of “Did not finish primary school and home school”, “Elementary school”, “Middle school and above” had higher BMI scores. The coefficients are 0.146 (SE = 0.089; *p* = 0.1), 0.287 (SE = 0.087; *p* = 0.001), and 0.527 (SE = 0.085; *p* < 0.001), respectively. That is, the higher the education level, the higher the BMI scores. Married couples significantly increased the BMI score, and the BMI score of the married respondents was 0.219 (SE = 0.087; *p* = 0.012) higher than that of unmarried respondents. That is, keeping other factors constant, the body weight would also increase gradually in the wedded life.

Concerning health behavior, an interesting conclusion was that only those who smoke displayed significant effects on BMI. Compared with non-smoking respondents, the BMI score of the smokers was reduced by 1.183 (SE = 0.075; *p* < 0.001). Although drinking wine also had negative effect on body weight, the impact was not significant even at 10% significant level for both regression model and m-logit model. Furthermore, we illustrated the impact of exercises on body weight. The amount of time spending on physical activity (exercise or work which is hard enough to make you breathe more heavily and to make your heart beat faster) in a usual week was used to measure respondent’s exercise level for at least 10 min. Examples include aerobics, fast bicycling, brisk walking, and heavy labor, e.g., heavy lifting, digging, plowing. Three levels of physical activity were coded as 0 times/wk, 1 to 3 times/wk, and 4 times/wk. The three levels of physical activity were also named as sedentary, moderately active, and very active. The results indicated that exercise had negative effect on body weight. The coefficient for moderately active exercise and very active exercise are −0.28 (SE = 0.142; *p* = 0.048) and −0.663 (SE = 0.096; *p* < 0.001), respectively. That is, the more exercise the people do, the thinner they are. As only 6505 respondents were asked the questions about the time of physical activity, we put the results including exercise in the Appendix A.

Table 4 depicts the effects of AQI on BMI according to WHO and Asian criteria. We divided the BMI values based on the WHO and Asian classification standards, respectively, and we obtained similar results to Table 3 in which we used Chinese criteria.

We changed the AQI calculation method and used the median AQI for the month to calculate the AQI indicator. Table 5 depicts the effects of the median AQI on BMI. With an increase in AQI, the BMI score increased by 0.03 (SE = 0.002; *p* < 0.001). Specifically, obese participants had a closer positive correlation than those who were underweight and overweight. The coefficient of “Underweight” Chinese criteria BMI scores was 1.0, and a BMI of “Normal”, “Overweight”, and “Obese” were more likely to be reported by 1 percent (95% CI: 1.005, 1.015), 2.3 percent (95% CI: 1.018, 1.029) and 3.1 percent (95% CI: 1.026, 1.037), respectively. Although we changed the AQI calculation method and we obtained similar results to Table 3.

The effect of AQI on BMI with categorical AQI levels is shown in Table 6. Each categorical AQI level has significantly positive effects on the BMI score. Compared with excellent air quality (0–49.9), the influence of good air quality (50–99.9) and mild pollution (100–149.9) on BMI score is 0.752 (SE = 0.069; *p* < 0.001) and 1.364 (SE = 0.207; *p* < 0.001). Under the same pollution level, the influence of AQI increased as the obesity of participants increased, for example, AQI belonged to 50–99.9.

Effect of AQI on BMI with AQI lag is shown in Table 7. The results reveal a significantly positive relationship between each lagged AQI and the BMI score. When we used a one-month lag, the BMI score increased by 0.027 (SE = 0.001; *p* < 0.001) with AQI growth. When we used a two-month lag, with AQI increasing, the BMI score increased by 0.022 (SE = 0.001; *p* < 0.001). When we used a three-month lag, the BMI score increased by 0.024 (SE = 0.001; *p* < 0.001).

From Table 3; Table 7, we found that both the current AQI and the lagged AQI (one-month lag, two-month lag and three-month lag) had a positive effect on BMI. However, the impact from AQI gradually decreased. That is, the farther away from the month of the interview, the smaller the impact.

Effects of other air pollution on BMI are shown in Table 8. All other air pollutants (PM_2.5_, PM_10_, CO, NO_2_, O_3_ and SO_2_) represent a significantly positive impact on the BMI score. When one unit of PM_2.5_, PM_10_, CO, NO_2_, O_3_, or SO_2_ was added, the BMI score increased by 0.034 (SE = 0.002; *p* < 0.001), 0.023 (SE = 0.001; *p* < 0.001), 0.52 (SE = 0.095; *p* < 0.001), 0.045 (SE = 0.004; *p* < 0.001), 0.021 (SE = 0.002; *p* < 0.001), 0.008 (SE = 0.003; *p* = 0.015), respectively. We found that CO (the coefficient: 0.52 (SE = 0.095; *p* < 0.001)) had relatively larger impacts than other air pollutants. That is, from the perspective of the one-unit air pollution increase, the order of the impact for the six pollutants from large to small is: CO, NO_2_, PM_2.5_, PM_10_, O_3_, and SO_2_, respectively.

Table 9 reports the effect of AQI on BMI from the perspective of sex, age, and education. We split the samples by sex, age, and education, respectively, and obtained results similar to Table 5 regarding the effects of AQI on the BMI score. All air pollution consistently led to an increase in the BMI score. Additionally, from the results of the OLS model, females were more susceptible to AQI in terms of obesity than males were (0.034 (SE = 0.002; *p* < 0.001) vs. 0.027 (SE = 0.002; *p* < 0.001)). There was no significant difference for young people and elderly people in the OLS regression analysis (0.03 (SE = 0.002; *p* < 0.001) vs. 0.03 (SE = 0.002; *p* < 0.001)), while in the m-logit model, young people were more susceptible to AQI than the elderly (1.015 (95%: 1.005, 1.025) vs. 1.008 (95%: 1.002, 1.014) for normal weight; 1.027 (95%: 1.017, 1.037) vs 1.023 (95%: 1.017, 1.03) for overweight and 1.036 (95%: 1.026, 1.047) vs 1.031 (95%: 1.024, 1.038) for obese). That is, age had much greater influence on body weight in young people, and people lower than 60 should pay more attention to body weight. For education levels, the higher the level of education, the less the BMI was affected by AQI (0.037 (SE = 0.003; *p* < 0.001) vs. 0.029 (SE = 0.003; *p* < 0.001) vs. 0.027 (SE = 0.003; *p* < 0.001)). The results estimated by the m-logit method were consistent with the OLS results. The difference was that the age group test that indicated the impact of pollution on middle-aged people was greater than that of the elderly.

## 4. Discussion

The 2015 CHARLS information and air pollution data for 125 cities from a national urban air quality monitoring network were used with ordinary least squares (OLS) and multinomial logit model (m-logit) estimation to assess the impact of air pollution on obesity in China. We chose different air pollution and BMI indicators for estimation and examined the impact of lagging air pollution. The similarity in results indicates a relatively stable relationship. We also used sex, age, and academic characteristics to stratify the samples and study their impact on the relationship between air pollution and obesity. As expected, air pollution can induce obesity, and the higher the degree of air pollution, the more serious is the effect on obesity. The closer to the survey month, the more serious the impact; it can be concluded that pollution has a cumulative effect on health. In addition, we found that CO is the most influential pollutant; we must, therefore, pay special attention to the changes in their concentration. We also identified that women, the middle-aged, and those with low levels of education were especially susceptible [22]. In the Public Health and Air Pollution in Asia (PAPA) study, Kan et al. (2008) [38] found that women were more susceptible to the effects of air pollution. In a pooled study of 130,000 respiratory deaths in 27 US. communities, Franklin et al. (2007) [39] revealed that community air pollution better predicted death among women than among men. Sex-specific lifestyle explanations (increased smoking among men may obscure pollution effects) and biological explanations (females’ smaller airways and higher airway reactivity) [40] might partially account for these effects. Although we observed that the effect of air pollution exposure on obesity was evident in females, additional inquiry into why this is the case is needed. Additionally, when stratified by age, the results showed that air pollution and obesity were more strongly correlated among older participants, especially for the prevalence of obesity, suggesting that older subgroups may be more sensitive to ambient air pollutants [26].

Our research adds evidence to the relationship between air pollution and obesity, which could provide important recommendations to minimize the risk of obesity due to air pollution. To avoid obesity, we should try our best to live in an environment with good air quality. Our findings provide significant evidence for people to choose an urban life, as high concentrations of air pollution could lead to health risks such as obesity. In daily life, residents should take necessary measures to reduce health risks from air pollution, such as wearing a mask, avoiding outdoor sports on heavy pollution days, using an air purifier, and placing green plants indoors. Past air pollution still has an impact on obesity, and the longer the lag period, the weaker the impact, relative to the current period. This means that air pollution will accumulate in the human body. Furthermore, identifying susceptible populations helps the government to formulate health policies.

It is necessary to discuss the limitations of the current study. First, genetics is an important factor that affects obesity [41,42]. Due to data limitations, we could not calculate the contribution from genetics on obesity. Further, because air pollution data at the city level were launched only in 2014, this study used a cross-sectional design and the findings cannot be applied to demonstrate a causal relationship between long-term air pollution and obesity. In the future, with the enrichment of micro-individual and air pollution data, we could use panel data to improve our analysis.

## 5. Conclusions

This study evaluated the relationship between air pollution and obesity based on data from the Chinese middle-aged and elderly populations. It found that air pollution is an important factor affecting obesity: the higher the air pollution level, the more serious the impact, and this impact has a cumulative effect. Additionally, CO is the most influential pollutant and women, the middle-aged, and those with low levels of education are the most severely affected. Therefore, when developing public health policies, the adverse health effects of air pollution should be considered.

## Figures and Tables

**Table 1:** BMI according to WHO, Asian and Chinese criteria.

	Chinese Criteria	WHO Criteria	Asian Criteria
Underweight	<18.5	<18.5	<18.5
Normal	18.5–23.9	18.5–24.9	18.5–22.9
Overweight	24–27.9	25–29.9	23–24.9
Obese	≥28	≥30	≥25

**Table 2:** Characteristics of the study population.

	Full Sample	BMI According to Chinese Criteria			
	(*N* = 13,414)	BMI < 18.5	18.5 ≤ BMI < 23.9	24 ≤ BMI < 27.9	BMI ≥ 28
		(*N* = 700)	(*N* = 6651)	(*N* = 4451)	(*N* = 1612)
	Frequency	Frequency	Frequency	Frequency	Frequency
Male	6264	348	3413	1918	585
Married	11,622	563	5662	3949	1448
Uninsured	1089	70	562	345	112
Education					
No Formal Education	3447	257	1793	998	399
Did not Finish Primary School and Home School	2641	153	1396	795	297
Elementary School	3042	143	1519	1028	352
Middle School and Above	4284	147	1943	1630	564
Smoking	4127	288	2406	1136	297
Drinking	3500	171	1892	1111	326
**Categorical BMI Levels**					
BMI < 18.5	700	700	0	0	0
18.5 ≤ BMI < 24	6651	0	6651	0	0
24 ≤ BMI < 28	4451	0	0	4451	0
BMI ≥ 28	1612	0	0	0	1612
**Categorical AQI Levels**					
AQI < 49.9	2790	196	1558	801	235
50 < AQI ≤ 99.9	10,316	491	4970	3542	1313
100 < AQI ≤ 149.9	308	13	123	108	64
	Mean (SD)	Mean (SD)	Mean (SD)	Mean (SD)	Mean (SD)
Age	60.932 (9.908)	66.556 (10.762)	61.794 (10.071)	59.638 (9.295)	58.51 (9.084)
BMI	23.767 (3.45)	17.72 (0.535)	21.586 (1.496)	25.758 (1.122)	29.89 (1.511)
**Continuous AQI**					
Interview Month	64.391 (17.656)	59.726 (16.905)	62.29 (16.892)	66.525 (17.851)	69.193 (18.71)
One-Month Lag	67.104 (22.311)	60.322 (20.102)	64.261 (21.25)	69.887 (22.674)	74.097 (23.698)
Two-Month Lag	74.038 (22.811)	68.243 (21.767)	71.573 (22.424)	76.488 (22.815)	79.956 (22.849)
Three-Month Lag	79.716 (20.948)	74.615 (19.843)	77.42 (20.015)	81.927 (21.445)	85.3 (21.941)
**Other Air Pollution**					
PM_2.5_	37.655 (14.121)	34.676 (13.29)	36.117 (13.365)	39.184 (14.516)	41.069 (15.25)
PM_10_	66.193 (24.413)	59.764 (22.35)	63.12 (22.968)	69.264 (25.153)	73.183 (26.24)
CO	0.837 (0.306)	0.794 (0.261)	0.825 (0.296)	0.847 (0.316)	0.875 (0.333)
NO_2_	23.14 (8.201)	21.684 (8.006)	22.508 (8.13)	23.775 (8.209)	24.632 (8.2)
O_3_	67.888 (16.914)	65.07 (16.215)	66.661 (16.846)	69.057 (16.78)	70.948 (17.2)
SO_2_	16.099 (9.109)	16.122 (9.028)	15.884 (9.01)	16.374 (9.309)	16.213 (8.976)

**Table 3:** Effect of Air Quality Index (AQI) on Body Mass Index (BMI).

Variables	The BMI Score		BMI According to Chinese Criteria					
			Normal		Overweight		Obese	
	Coef.	Std. Err.	Adjusted OR	95% CI	Adjusted OR	95% CI	Adjusted OR	95% CI
AQI	0.031 ***	0.002	1.01 ***	1.005, 1.015	1.024 ***	1.019, 1.029	1.032 ***	1.027, 1.038
Male	−0.229 ***	0.078	1.32 **	1.06, 1.645	1.106	0.881, 1.388	1.091	0.847, 1.406
Age	−0.052 ***	0.003	0.954 ***	0.945, 0.962	0.938 ***	0.929, 0.946	0.924 ***	0.914, 0.933
Education								
Did Not Finish Primary School and Home School	0.146 *	0.089	1.114	0.891, 1.392	1.198	0.948, 1.514	1.123	0.861, 1.464
Elementary School	0.287 ***	0.087	1.225 *	0.971, 1.546	1.548 ***	1.216, 1.97	1.302 *	0.992, 1.708
Middle School and Above	0.527 ***	0.085	1.241 *	0.974, 1.581	1.826 ***	1.443, 2.343	1.488 ***	1.13, 1.96
Married	0.219 **	0.087	0.831 *	0.666, 1.036	0.949	0.752, 1.198	0.984	0.75, 1.293
Uninsured	−0.049	0.103	1.004	0.768, 1.313	1.013	0.765, 1.341	0.908	0.657, 1.257
Smoking	−1.183 ***	0.075	0.612 ***	0.5, 0.75	0.391 ***	0.316, 0.484	0.279 ***	0.218, 0.357
Drinking	−0.062	0.07	1.16	0.95, 1.416	1.141	0.927, 1.404	0.99	0.781, 1.256
Constant	24.989 ***	0.266	110.851 ***	52.671, 233.293	79.481 ***	36.842, 171.468	49.531 ***	21.065, 116.466
R-Squared	0.092		0.039					
Log Likelihood			–14,466					

Notes: *N* = 13,414. * *p* < 0.10; ** *p* < 0.05; *** *p* < 0.01.

**Table 4:** Effect of Air Quality Index (AQI) on Body Mass Index (BMI): BMI according to WHO and Asian criteria.

Variables	BMI According to Chinese Criteria					
	Normal		Overweight		Obese	
	Adjusted OR	95% CI	Adjusted OR	95% CI	Adjusted OR	95% CI
**A. BMI According to WHO Criteria**						
AQI	1.012 ***	1.007, 1.017	1.026 ***	1.021, 1.032	1.036 ***	1.029, 1.043
R-Squared	0.04					
Log Likelihood	−12,369					
**B. BMI According to Asian Criteria**						
AQI	1.008 ***	1.003, 1.013	1.02 ***	1.014, 1.025	1.028 ***	1.023, 1.033
R-Squared	0.038					
Log Likelihood	−15,733					

Notes: *N* = 13,414. *** *p* < 0.01.

**Table 5:** Effect of Air Quality Index (AQI) on Body Mass Index (BMI): median AQI.

Variables	The BMI Score		BMI According to Chinese Criteria					
			Normal		Overweight		Obese	
	Coef.	Std. Err.	Adjusted OR	95% CI	Adjusted OR	95% CI	Adjusted OR	95% CI
AQI	0.03 ***	0.002	1.01 ***	1.005, 1.015	1.023 ***	1.018, 1.029	1.031 ***	1.026, 1.037
R-Squared	0.091		0.039					
Log Likelihood			−14,471					

Notes: *N* = 13,414. *** *p* < 0.01. AQI is constructed using median AQI for the month.

**Table 6:** Effect of Air Quality Index (AQI) on Body Mass Index (BMI): categorical AQI levels.

Variables	The BMI Score		BMI According to Chinese Criteria					
			Normal		Overweight		Obese	
	Coef.	Std. Err.	Adjusted OR	95% CI	Adjusted OR	95% CI	Adjusted OR	95% CI
50 < AQI ≤ 99.9	0.752 ***	0.069	1.296 ***	1.085, 1.548	1.799 ***	1.492, 2.168	2.336 ***	1.872, 2.914
100 < AQI ≤ 149.9	1.364 ***	0.207	1.215	0.67, 2.206	2.049 **	1.117, 3.758	4.24 ***	2.238, 8.03
R-squared	0.076		0.033					
Log likelihood			−14,556					

Notes: *N* = 13,414. ** *p* < 0.05; *** *p* < 0.01.

**Table 7:** Effect of Air Quality Index (AQI) on Body Mass Index (BMI): AQI lag.

Variables	The BMI Score		BMI According to Chinese Criteria					
			Normal		Overweight		Obese	
	Coef.	Std. Err.	Adjusted OR	95% CI	Adjusted OR	95% CI	Adjusted OR	95% CI
One-Month Lag	0.027 ***	0.001	1.01 ***	1.006, 1.014	1.022 ***	1.017, 1.026	1.03 ***	1.025, 1.034
R-Squared	0.098		0.042					
Log Likelihood			−14,433					
Two-Month Lag	0.022 ***	0.001	1.007 ***	1.003, 1.01	1.016 ***	1.012, 1.02	1.023 ***	1.019, 1.027
R-Squared	0.088		0.038					
Log Likelihood			−14,491					
Three-Month lag	0.024 ***	0.001	1.008 ***	1.003, 1.012	1.018 ***	1.014, 1.022	1.025 ***	1.02, 1.03
R-Squared	0.089		0.038					
Log Likelihood			−14,489					

Notes: *N* = 13,414. *** *p* < 0.01. AQI is constructed using the median 1–3 month prior to the interview.

**Table 8:** Effect of other air pollution on Body Mass Index (BMI).

Variables	The BMI Score		BMI According to Chinese Criteria					
			Normal		Overweight		Obese	
	Coef.	Std. Err.	Adjusted OR	95% CI	Adjusted OR	95% CI	Adjusted OR	95% CI
PM_2.5_	0.034 ***	0.002	1.009 ***	1.003, 1.016	1.025 ***	1.018, 1.032	1.034 ***	1.027, 1.041
R-Squared	0.087		0.037					
Log Likelihood			−14,498					
PM_10_	0.023 ***	0.001	1.007 ***	1.003, 1.011	1.017 ***	1.013, 1.021	1.024 ***	1.019, 1.028
R-Squared	0.093		0.04					
Log Likelihood			−14,458					
CO	0.52 ***	0.095	1.385 **	1.036, 1.851	1.631 ***	1.213, 2.193	2.145 ***	1.56, 2.949
R-Squared	0.07		0.03					
Log Likelihood			−14,602					
NO_2_	0.045 ***	0.004	1.013 **	1.003, 1.024	1.032 ***	1.022, 1.043	1.046 ***	1.034, 1.058
R-Squared	0.079		0.034					
Log Likelihood			−14,552					
O_3_	0.021 ***	0.002	1.006 **	1.001, 1.011	1.015 ***	1.01, 1.02	1.022 ***	1.016, 1.028
R-Squared	0.078		0.033					
Log Likelihood			−14,555					
SO_2_	0.008 **	0.003	0.999	0.99, 1.007	1.005	0.996, 1.014	1.003	0.993, 1.013
R-Squared	0.068		0.03					
Log Likelihood			−14615					

Notes: *N* = 13,414. ** *p* < 0.05; *** *p* < 0.01.

**Table 9:** Effect of Air Quality Index (AQI) on Body Mass Index (BMI): by sex, age, and education.

Variables	The BMI Score		BMI According to Chinese Criteria					
			Normal		Overweight		Obese	
	Coef.	Std. Err.	Adjusted OR	95% CI	Adjusted OR	95% CI	Adjusted OR	95% CI
**A. Sex**								
Male (*N* = 6264)								
AQI	0.027 ***	0.002	1.012 ***	1.005, 1.019	1.026 ***	1.018, 1.034	1.032 ***	1.024, 1.041
R-Squared	0.104		0.047					
Log Likelihood			–6420					
Female (*N* = 7150)								
AQI	0.034 ***	0.002	1.009 **	1.002, 1.016	1.023 ***	1.015, 1.03	1.033 ***	1.024, 1.04
R-Squared	0.064		0.028					
Log Likelihood			−8012					
**B. Age**								
Age < 60 (*N* = 7271)								
AQI	0.03 ***	0.002	1.015 ***	1.005, 1.025	1.027 ***	1.017, 1.037	1.036 ***	1.026, 1.047
R-Squared	0.056		0.023					
Log Likelihood			−6689					
Age ≥ 60 (*N* = 6143)								
AQI	0.03 ***	0.002	1.008 ***	1.002, 1.014	1.023 ***	1.017, 1.03	1.031 ***	1.024, 1.038
R-Squared	0.097		0.041					
Log Likelihood			−7748					
**C. Education**								
Edu = 1 or 2 (*N* = 6182)								
AQI	0.037 ***	0.003	1.009 ***	1.002, 1.015	1.025 ***	1.018, 1.032	1.034 ***	1.026, 1.041
R-Squared	0.092		0.046					
Log Likelihood			−6774					
Edu = 4 (*N* = 3042)								
AQI	0.029 ***	0.003	1.01 *	0.999, 1.022	1.025 ***	1.013, 1.037	1.031 ***	1.018, 1.044
R-Squared	0.097		0.041					
Log Likelihood			−3229					
Edu = 5 (*N* = 4284)								
AQI	0.027 ***	0.003	1.012 **	1.001, 1.023	1.023 ***	1.012, 1.034	1.033 ***	1.021, 1.044
R-Squared	0.06		0.026					
Log Likelihood			−4628					

Notes: * *p* < 0.10; ** *p* < 0.05; *** *p* < 0.01.

## Appendix A

**Table A1:** Effect of Air Quality Index (AQI) on Body Mass Index (BMI): add physical activity variable.

Variables	The BMI Score		BMI According to Chinese Criteria					
			Normal		Overweight		Obese	
	Coef.	Std. Err.	Adjusted OR	95% CI	Adjusted OR	95% CI	Adjusted OR	95% CI
AQI	0.027 ***	0.002	1.006 *	0.999, 1.013	1.018 ***	1.011, 1.026	1.027 ***	1.019, 1.035
Male	−0.088	0.113	1.751 **	1.25, 2.452	1.434 **	1.014, 2.027	1.565 **	1.071, 2.288
Age	−0.055 ***	0.005	0.952 ***	0.939, 0.965	0.936 ***	0.923, 0.949	0.92 ***	0.905, 0.934
Education								
Did Not Finish Primary School and Home School	0.195	0.126	1.269	0.908, 1.773	1.406 *	0.992, 1.993	1.405 *	0.951, 2.076
Elementary School	0.348 ***	0.123	1.252	0.892, 1.759	1.715 ***	1.208, 2.436	1.434 *	0.968, 2.124
Middle School and Above	0.475 ***	0.122	1.17	0.822, 1.665	1.747 ***	1.216, 2.511	1.409 *	0.945, 2.099
Married	0.303 **	0.13	0.934	0.672, 1.297	1.101	0.779, 1.556	1.016	0.681, 1.514
Uninsured	−0.176	0.146	1.263	0.821, 1.942	1.185	0.758, 1.852	0.986	0.592, 1.643
Smoking	−1.312 ***	0.107	0.562 ***	0.413, 0.764	0.327 ***	0.237, 0.450	0.218 ***	0.151, 0.314
Drinking	−0.011	0.099	1.158	0.856, 1.566	1.247	0.912, 1.704	1.099	0.774, 1.561
Moderately Active	−0.28 **	(0.142)	0.814	0.541, 1.224	0.716	0.470, 1.091	0.712	0.446, 1.136
Very Active	−0.663 ***	(0.096)	0.757 **	0.576, 0.994	0.57 ***	0.430, 0.757	0.507 ***	0.369, 0.697
Constant	25.495 ***	0.388	143.007 ***	45.414, 450.323	127.245 ***	39.133, 413.766	96.041 ***	26.265, 351.186
R-Squared	0.097		0.043					
Log Likelihood			−6994					

Notes: *N* = 6505. * *p* < 0.10; ** *p* < 0.05; *** *p* < 0.01.
